# Immunoregulation of Bone Marrow-Derived Mesenchymal Stem Cells on the Chronic Cigarette Smoking-Induced Lung Inflammation in Rats

**DOI:** 10.1155/2015/932923

**Published:** 2015-11-18

**Authors:** Xiaoyan Li, Junyan Wang, Jing Cao, Lijuan Ma, Jianying Xu

**Affiliations:** ^1^The First Clinical Medical College, Shanxi Medical University, Taiyuan 030001, China; ^2^Department of Respiratory Medicine, Shanxi DAYI Hospital of Shanxi Medical University, Taiyuan 030032, China

## Abstract

Impact of bone mesenchymal stem cell (BMSC) transfusion on chronic smoking-induced lung inflammation is poorly understood. In this study, a rat model of smoking-related lung injury was induced and the rats were treated with vehicle or BMSCs for two weeks. Different subsets of CD4+ T cells, cytokines, and anti-elastin in the lungs as well as the lung injury were characterized. Serum and lung inducible nitric oxide synthase (iNOS) and STAT5 phosphorylation in lymphocytes from lung tissue were also analyzed. Results indicated that transfusion of BMSCs significantly reduced the chronic smoking-induced lung injury, inflammation, and levels of lung anti-elastin in rats. The frequency of Th1 and Th17 cells and the levels of IL-2, IL-6, IFN-*γ*, TNF-*α*, IL-17, IP-10, and MCP-1 increased, but the frequency of Tregs and IL-10 decreased. Transfusion of BMSCs significantly modulated the imbalance of immune responses by mitigating chronic smoking-increased Th1 and Th17 responses, but enhancing Treg responses in the lungs of rats. Transfusion of BMSCs limited chronic smoking-related reduction in the levels of serum and lung iNOS and mitigated smoking-induced STAT5 phosphorylation in lymphocytes from lung tissue. BMSCs negatively regulated smoking-induced autoimmune responses in the lungs of rats and may be promising for the intervention of chronic smoking-related lung injury.

## 1. Introduction

Chronic obstructive pulmonary disease (COPD) is an obstructive disease of the lungs and characterized by chronic inflammatory lung damage, leading to progressive limitation of airflow. COPD is a major public health problem and affects many people in the world [1]. Long-term cigarette smoking is a risk factor of the development of COPD. However, the mechanisms by which chronic cigarette smoking causes the lung inflammation and damages leading to COPD have not been clarified.

The pathogenesis of chronic smoking-related COPD is complex. Chronic smoking-related inflammation and continual repairs and remodeling destroy the lung parenchyma and the alveolar tissues, leading to emphysema and obstruction of small airways. Smoking can induce oxidative stress and inflammation, which downregulate inducible nitric oxide synthase (iNOS) expression, and release proinflammatory cytokines and chemokines, recruiting inflammatory infiltrates, including neutrophils, macrophages, and lymphocytes in the lung. T cell autoimmunity may contribute to the development of chronic smoking-related pulmonary inflammation and COPD [2]. The numbers of proinflammatory T cells in the lungs of COPD patients are associated with the pathological degrees of COPD [3]. Chronic smoking-related inflammation can induce IFN-*γ*-secreting Th1 and IL-17A-secreting Th17 responses but inhibit anti-inflammatory IL-10-secreting regulatory T cell (Treg) responses, leading to an imbalance of T cell immunity. In addition, chronic smoking induces high levels of anti-elastin antibodies [4]. However, the frequency of functional CD4+ T cells in the lungs of individuals with COPD particularly during the early process of chronic smoking-related emphysema and their cytokine and chemokine production in the lungs have not been systemically investigated.

Bone marrow mesenchymal stem cells (BMSCs) are potent regulators of inflammation. BMSCs can secrete anti-inflammatory prostaglandin E_2_ (PGE_2_), kynurenine, IL-10, tumor necrosis factor- (TNF-) stimulated gene 6 protein (TSG-6), NO, and transforming growth factor (TGF)-*β*1, and inhibiting proinflammatory responses [5]. BMSCs can interact with effector cells, through the notch and Fas, and promote alternatively activated M2 cell maturation [6]. Transfusion of BMSCs can inhibit the progression of autoimmune diseases, including systemic sclerosis [7]. Accordingly, we hypothesized that transfusion of BMSCs could reduce chronic smoking-induced lung inflammation by modulating proinflammatory CD4+ T cell infiltration and limiting anti-elastin responses.

In this study, we employed a rat model of chronic cigarette smoking-induced inflammation and emphysema to test the impact of BMSCs on the lung inflammation and injury, CD4+ T cell infiltration in the lung, and humoral responses to elastin.

## 2. Materials and Methods

### 2.1. Animals and Cigarette Smoking

Male Sprague Dawley (SD) rats (8-week-old, weighing about 120 g) were from the Experimental Animal Center of Shanxi Medical University, China. The rats were housed in a specific pathogen-free facility. The experimental protocol was approved by the Animal Care and Research Committee of Shanxi Medical University.

Rats (*n* = 15 per group) were placed in an organic glass passive smoking cage (100 × 80 × 60 mm) and exposed to cigarette smoking of 20 filtered commercial cigarettes (the red canal brand cigarette, China) one hour, twice per day, 6 days per week for 24 weeks [8]. The control group animals were exposed to regular air. In this case, individual rats were exposed to 11 mg tar and 0.9 mg nicotine each time.

### 2.2. BMSC Isolation, Culture, and Identification

BMSCs were isolated from six- to eight-week-old male SD rats, as described previously [9]. Briefly, individual rats were injected intraperitoneally (i.p) with 25% urethane, and their femurs and tibias were excised. Their bone marrow cells were isolated by flushing the bone marrow cavity with complete medium (containing 10% fetal bovine serum). After being washed, the isolated bone marrow cells were cultured in complete medium at 37°C in a humidified atmosphere of 5% CO_2_ for three days. The unattached cells were removed and the adhered cells were continually cultured until 90% confluence. The cells were trypsinized and passaged at a ratio of 1 : 2 or 1 : 3. The third-passage BMSCs were pooled and used for characterization and treatment.

The harvested BMSCs were incubated with anti-CD16/32 on ice for 30 minutes and stained with FITC-anti-CD34, PE-anti-CD45, and FITC-anti-CD90 (BD Biosciences, San Jose, USA). The isotype control IgG served as negative controls. After being washed, BMSCs were analyzed by flow cytometry on a FACSCalibur flow cytometer and analyzed using the CellQuest analysis program (BD Biosciences). The BMSCs with purity of >90% were used for the treatment.

### 2.3. Treatment

At the end of the cigarette smoking (CS), the rats were randomly treated intravenously with 0.5 mL PBS as the CS+PBS group or with BMSCs (2 × 10^6^ cells in 0.5 mL) weekly for two weeks as the CS+BMSCs group. The control rats received the same volume of PBS.

### 2.4. Tissue Preparation and Histological Analysis

Individual rats were anesthetized i.p. with 25% urethane (800 mg/kg) and perfused with 50 mL PBS at the end of week 26. The middle lobe of right lung was inflated by intratracheal infusion of 0.3% low-melting agar at 25 cm H_2_O, fixed with 4% paraformaldehyde, and paraffin-embedded. The remaining right lung was frozen and stored at −80°C. The lung sections (4 *μ*m) were stained with hematoxylin and eosin (H&E). The mean linear intercept (MLI) and the mean alveolar numbers (MAN) per unit area were determined in a blinded manner.

### 2.5. Preparation of Lung Tissue Lymphocytes and Flow Cytometry

The left lung of individual rats was removed, cut into small pieces, digested with collagenase, and filtered through a 200-mesh sieve. After centrifugation, the collected single cell suspensions were subjected to density gradient centrifugation using Ficoll-Paque to isolate lymphocytes. Some of the collected lymphocytes were stimulated with 50 ng/mL phorbol 12-myristate 12-acetate (PMA) and 1 *μ*g/mL ionomycin in the presence of brefeldin A for 5 h (BD Biosciences, USA). The cells were stained with FITC-anti-CD4, fixed, and permeabilized. Subsequently, the cells were stained with PE-anti-IL-17A, PE-anti-CD25, and APC-anti-Foxp3 (eBioscience, USA) and PE-anti-IL-4 or APC-anti-IFN-*γ* (BD Biosciences) for the analysis of the frequency of CD4+IL-17+ Th17, CD4+IFN-*γ*+ Th1, CD4+IL-4+ Th2, and CD4+CD25+Foxp3+ Tregs in total CD4+ T cells, respectively. The isotype rat Ig served as negative controls. After being washed, the stained cells were analyzed in flow cytometry in a FACSCalibur (BD FACSCanto II, USA) using CellQuest software.

The remaining lymphocytes were stained with PI using a DNA assay kit (KeyGEN Biotech, China). After being washed, the cells were analyzed for their cell cycling by flow cytometry.

### 2.6. Milliplex Protein Array System

The levels of cytokine and chemokine expression in the lung tissues were determined by a multiplex cytokine bead array using the MILLIPLEX Rat Cytokine/Chemokine 8-plex assay kit (Millipore, St. Charles, USA), according to the manufacturers' instructions. The reaction mixture was read using the Bio-Plex protein array reader, and the data were analyzed with the Bio-Plex Manager software program.

### 2.7. Western Blot Analysis and Enzyme-Linked Immunosorbent Assay (ELISA)

Antibodies to phosphorylated Stat5A/Stat5B (Millipore, USA), Stat5a (Abcam, Hong Kong) were used for Western blotting. The isolated lymphocytes from lung tissues were lysed on ice in a buffer consisting of 50 mM Tris (pH 7.5), 150 mM NaCl, 0.5% NP40, and complete proteinase inhibitor cocktail (Boster Biological Engineering, China). After being centrifuged, the cell lysates (30 *μ*g/lane) were separated by sodium dodecyl sulfate-polyacrylamide gel electrophoresis (SDS-PAGE) and transferred to a PVDF membrane (Boster). The membrane was blocked with 5% fat-free dry milk in TBST and incubated with antibodies against phosphorylated Stat5A/STAT5B (Millipore, USA), or Stat5a. The bound antibodies were detected using horseradish peroxidase- (HRP-) conjugated second antibodies and visualized using enhanced chemiluminescence reagents (Applygen Technologies, China). The relative levels of Stat5 phosphorylation in lymphocytes were determined by densitometry analysis.

The levels of lung anti-elastin antibodies and serum and lung iNOS were measured by ELISA using a specific kit (Ya'anda Biological Technology, Beijing, China), according to the manufacturers' protocol.

### 2.8. Statistical Analysis

Data are presented as the means ± SD. Difference between groups was analyzed by one-way analysis of variance (ANOVA) and post hoc LSD and Tamhane test using SPSS 13.0 software. A *p* value of <0.05 was considered statistically significant.

## 3. Results

### 3.1. BMSCs Mitigate Chronic Cigarette Smoking-Induced Lung Injury in Rats

**Figure 1:**
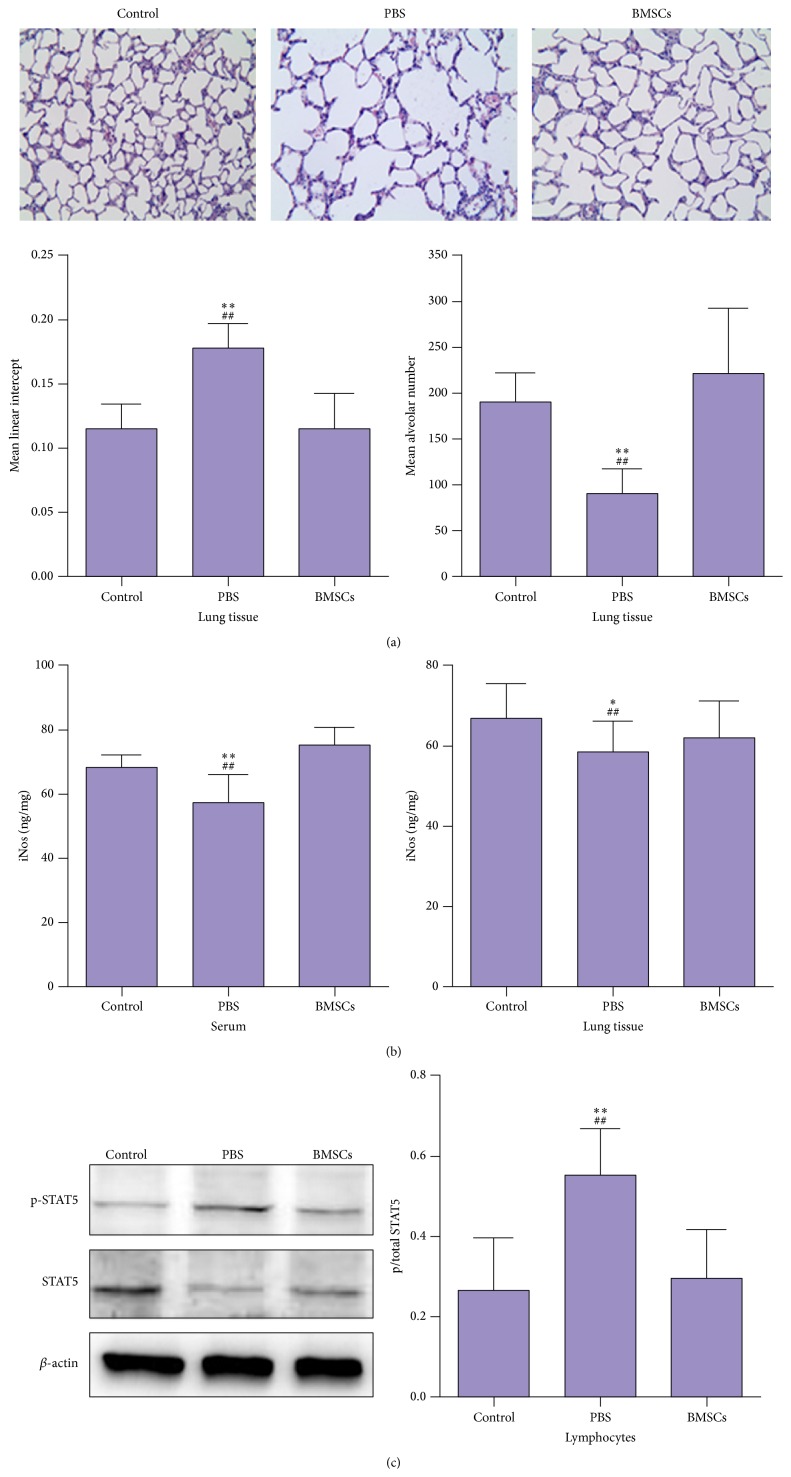
BMSCs mitigate the chronic cigarette smoking-induced lung injury, STAT5 phosphorylation, but they elevate iNOS expression in rats. The lung sections were stained with hematoxylin and eosin, and the mean linear intercept (MLI) and the mean alveolar numbers (MAN) per unit area in rats were determined (a). The levels of serum and lung iNOS (b) and STAT5 phosphorylation (c) were examined. Data are representative images (magnification ×400) and expressed as the means ± SD of individual group (*n* = 10 per group) from three separate experiments. ^*∗*^ *p* < 0.05, ^*∗∗*^ *p* < 0.01 versus the control; ^##^ *p* < 0.01 versus the BMSCs.

Following induction and treatment, the lung tissue sections were stained with H&E and the degrees of lung injury was evaluated by measuring the MLI and MAN. As shown in Figure 1(a), obviously enlarged alveolar cells and infiltrates were observed in the lungs of PBS-treated smoking group of rats, demonstrating chronic cigarette smoking-induced lung injury. Quantitative analyses indicated that the values of MLI and MAN in the BMSC-treated rats were similar to those of the healthy controls, and transfusion of BMSCs significantly reduced the values of MLI (0.11 ± 0.02 versus 0.18 ± 0.02, *p* < 0.01) but increased the values of MAN (221.18 ± 71.51 versus 91.12 ± 26.91, *p* < 0.01), related to the PBS-treated smoking rats.

We examined the impact of BMSCs on the smoking-related iNOS expression by ELISA. We found that the levels of serum and lung iNOS in the BMSC-treated rats were similar to those in the healthy controls but significantly higher than those in the PBS-treated smoking rats (*p* < 0.01, Figure 1(b)). We further examined the relative levels of STAT5 phosphorylation in lymphocytes from lung tissue. The relative levels of STAT5 phosphorylation in lymphocytes from the lungs of PBS-treated smoking rats were significantly higher than those in the healthy control and BMSC-treated smoking rats (*p* < 0.05) and there was no significant difference in the relative levels of STAT5 phosphorylation in these two groups of rats (Figure 1(c)).

### 3.2. BMSCs Mitigate Chronic Cigarette Smoking-Induced Anti-Elastin Responses in the Lungs of Rats

**Figure 2:**
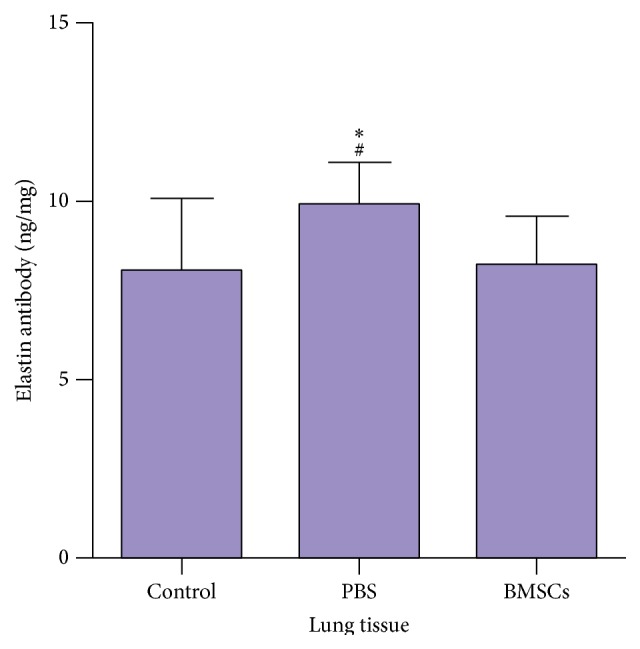
BMSCs mitigate the chronic smoking-induced anti-elastin responses in the lungs of rats. The levels of lung anti-elastin antibodies in rats were measured. Data are the means ± SD of individual groups of rats (*n* = 10 per group) from three separate experiments. ^*∗*^ *p* < 0.05 versus the control; ^#^ *p* < 0.05 versus the BMSCs.

We analyzed the levels of anti-elastin in the lung tissues by ELISA and found that the levels of anti-elastin antibodies in the lungs of PBS-treated smoking rats (9.89 ± 1.18 ng) were significantly higher than those in the healthy controls (8.06 ± 1.98 ng) and BMSC-treated smoking rats (8.25 ± 1.35 ng, Figure 2; *p* < 0.05 for both) and there was no significant difference between the BMSC-treated smoking rats and healthy controls.

### 3.3. BMSC Modulates Inflammatory Infiltrates in the Lungs of Smoking Rats

**Figure 3:**
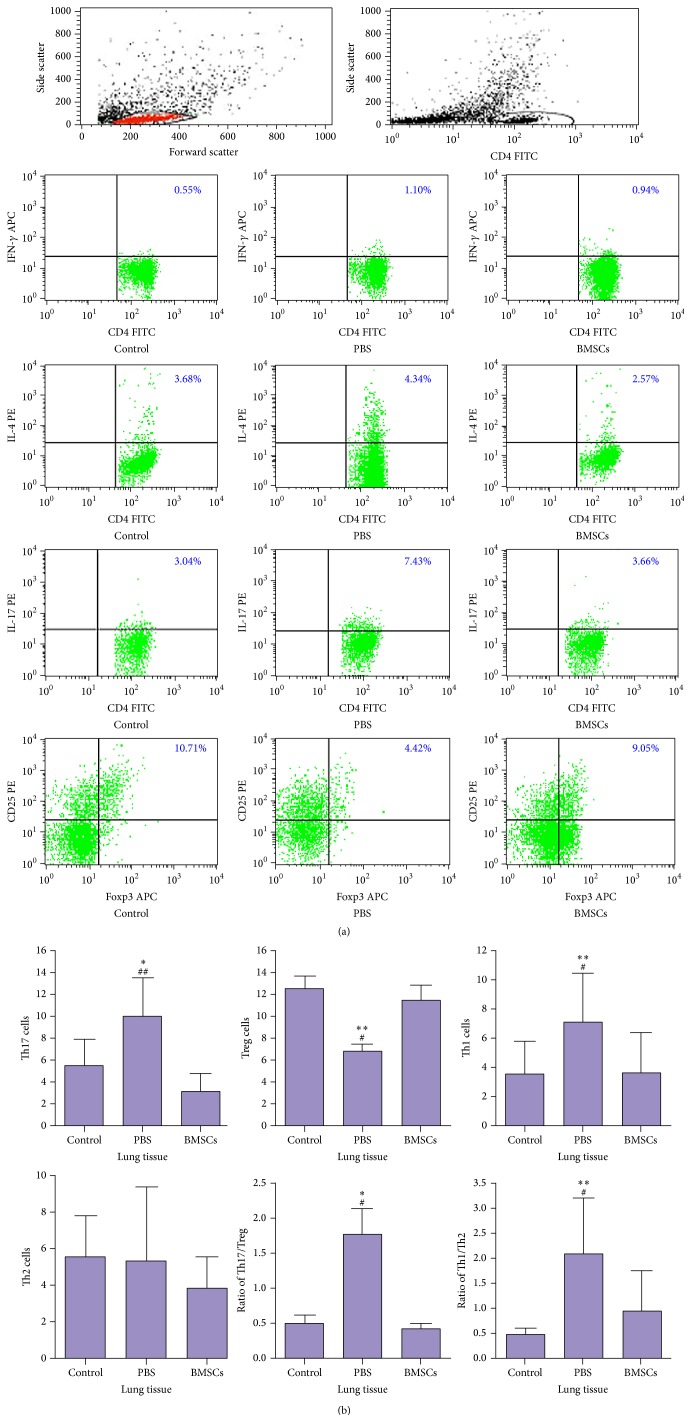
Flow cytometry analysis of the frequency of different subsets of CD4+ T cells in the lungs of rats. The lung-infiltrated lymphocytes were isolated from individual rats and stimulated with PMA/ionomycin for 5 hours. After being stained, the cells were first gated on lymphocytes and then gated on CD4+ T subsets. Subsequently, the percentages of CD4+IL-17+ Th17, CD4+CD25+Foxp3+ Tregs, CD4+IFN-*γ*+ Th1, and CD4+IL-4+ Th2 cells in total CD4+ T cells were analyzed. Data are representative FACS charts and expressed as the means ± SD of individual groups of rats (*n* = 10 per group) from four independent experiments. (a) Flow cytometry analysis. (b) Quantitative analysis. ^*∗*^ *p* < 0.05, ^*∗∗*^ *p* < 0.01 versus the controls; ^#^ *p* < 0.05, ^##^ *p* < 0.01 versus the BMSCs.

We characterized the frequency of different subsets of T cell infiltrates in the lungs of rats. There was no significant difference in the frequency of Th2 cells and different phases of CD4+ T cells in the lungs among different groups of rats (Figure 3). In comparison with that in the healthy controls, there was a significantly increased frequency of Th17, Th1 and lower frequency of Tregs in the lungs of PBS-treated smoking rats (*p* < 0.05 or *p* < 0.01). As a result, the ratios of Th17 to Tregs or Th1 to Th2 cells were also significantly greater in the lungs of PBS-treated smoking rats. In contrast, transfusion of BMSCs significantly reduced the frequency of Th17 and Th1 cells, but increased the frequency of Tregs in the lungs, related to the PBS-treated smoking rats (*p* < 0.05 or *p* < 0.01), which led to reduction in the ratios of Th17 to Tregs and Th1 to Th2 cells in the lungs.

### 3.4. BMSCs Modulate the Imbalance of Cytokine and Chemokines in the Lungs of Smoking Rats

**Figure 4:**
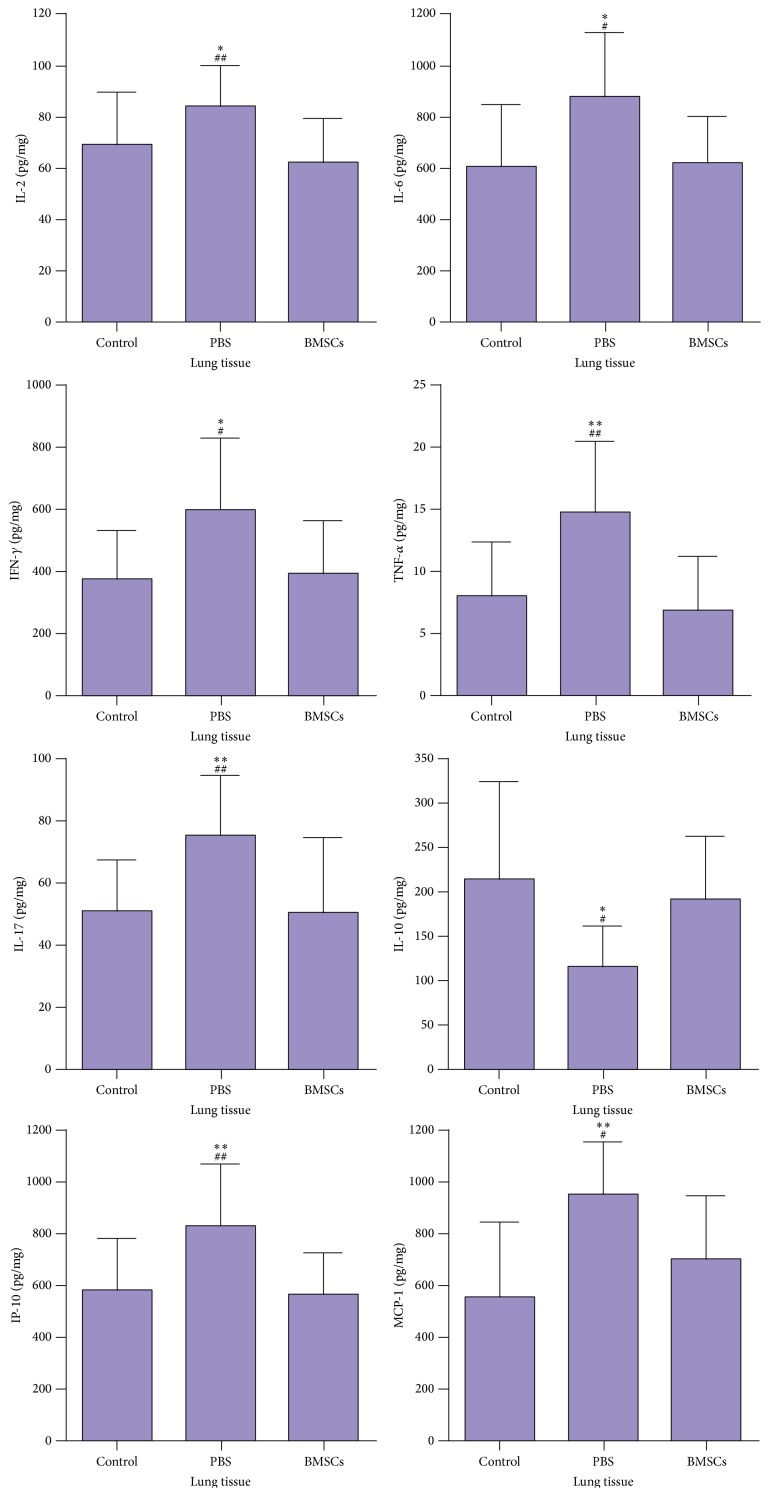
BMSCs modulate the cytokines/chemokines profile in the lungs of rats. The levels of lung cytokines/chemokines were measured. Data are the means ± SD per mg total proteins of individual groups of rats (*n* = 10 per group) from three separate experiments. ^*∗*^ *p* < 0.05, ^*∗∗*^ *p* < 0.01 versus the control; ^#^ *p* < 0.05, ^##^ *p* < 0.01 versus the BMSCs.

Finally, the levels of IP-10, MCP-1, IL-2, IL-6, IL-10, IFN-*γ*, TNF-*α*, and IL-17 in the lungs of rats were measured. In comparison with those in healthy controls, significantly higher levels of IP-10, MCP-1, IL-2, IL-6, IFN-*γ*, TNF-*α*, and IL-17, but lower levels of IL-10, were detected in the lungs of PBS-treated smoking rats (*p* < 0.05). In comparison with those in the PBS-treated smoking rats, the levels of IP-10, MCP-1, IL-2, IL-6, IFN-*γ*, TNF-*α*, and IL-17 in the lungs of BMSC-treated smoking rats were significantly reduced, but the levels of IL-10 in the lungs were significantly elevated (*p* < 0.05) (Figure 4).

## 4. Discussion

Chronic cigarette smoking induces lung inflammation and injury and is associated with the development of COPD. BMSCs are potent regulators of inflammation and have been demonstrated to inhibit autoimmune responses in several models of autoimmune diseases [7, 10]. Furthermore, transfusion of BMSCs has been sued for the treatment of patients with autoimmune diseases, such as systemic lupus erythematosus (SLE) and rheumatoid arthritis (RA) [11, 12]. In this study, we investigated the impact of BMSC transfusion on chronic smoking-induced lung inflammation and injury as well as autoimmune responses in rat model of chronic smoking-related pulmonary inflammation and injury. We found that chronic smoking induced significantly enlarged alveolar cells and lung injury, accompanied by increased values of MLI, but it decreased MAN in rats, consistent with a previous observation [13]. Transfusion of BMSCs significantly mitigated the smoking-induced lung inflammation and injury in rats. Furthermore, transfusion of BMSCs improved the imbalance of proinflammatory Th1, Th17, and anti-inflammatory Treg responses as well as anti-elastin responses in the lungs of rats. Some studies have indicated that NO inhibits the proliferation of T cells by suppressing the phosphorylation of signal transducer and activator of transcription-5 (STAT5), a transcription factor crucial for T cell activation and proliferation [14, 15]. Interestingly, transfusion of BMSCs mitigated the smoking-related reduction in the levels of serum and lung iNOS and reduced smoking-induced STAT5 activation in lymphocytes from lung tissue, which may be attributed to the secretion of high levels of iNOS by BMSCs and NO production. Our data were consistent with a previous observation and extended previous findings that transfusion with BMSCs inhibits autoimmune inflammation in mice [7, 16]. Our findings support the notion that BMSCs have potent inhibition on chronic smoking-induced inflammation and promote the pulmonary repair [17]. Therefore, our data extended previously suggest that BMSCs may be promising for the intervention of smoking-related pulmonary inflammation.

A recent study has indicated that autoimmune responses contribute to the pathogenesis of COPD [18]. It is well known that imbalance of proinflammatory Th1 and Th17 and anti-inflammatory Treg responses is crucial for the development of autoimmune diseases. We found that proinflammatory autoimmune responses may participate in the pathogenesis of smoking-related lung inflammation and injury in rats. Evidentially, in comparison with that in the healthy rats, significantly higher frequency of Th1, Th17, but reduced frequency of Tregs, was detected in the lungs of smoking rats, consistent with a previous report [18]. Second, significantly higher levels of IP-10, MCP-1, IL-2, IL-6, IFN-*γ*, TNF-*α*, and IL-17, but lower levels of IL-10, were detected in the lungs of smoking rats. Furthermore, significantly higher levels of lung anti-elastin antibodies and STAT5 phosphorylation were detected in smoking rats. It is possible that chronic smoking stimulates the production of proinflammatory chemokines, such as IP-10 and MCP-1 in the lungs, which recruit T cells, leading to the development and cascade of autoimmune responses and the lung injury. We are interested in further identifying antigen specificity of T cells.

Interestingly, we found that transfusion of BMSCs not only improved the smoking-related imbalance of proinflammatory Th1, Th17 and anti-inflammatory Tregs as well as their cytokines, but also mitigated the smoking-induced anti-elastin responses in the lungs of rats. Apparently, modulation of the smoking-related imbalance of proinflammatory T cell and anti-inflammatory Treg responses is crucial for the intervention of smoking-related lung inflammation. It is well known that BMSCs can secrete anti-inflammatory IL-10 and TGF-*β*1, which are important enhancers of functional Treg development [19]. Subsequently, functional Tregs can also inhibit proinflammatory Th1 and Th17 responses by direct cell-cell contact and also by indirectly secreted anti-inflammatory IL-10 and other inhibitory cytokines, creating a positive feedback to enhance inhibitory effect on inflammation [20]. In addition, BMSCs can differentiate into other types of cells and secrete other mediators for tissue repair, which may also contribute to their therapeutic effects we observed in the lungs of rats [21, 22].

Previous studies have shown that mouse BMSCs can secrete iNOS and human BMSCs secrete IDO, leading to the inhibition of T cell proliferation and induction of T cell energy [23, 24]. In this study, we found significantly lower levels of serum and lung iNOS in the smoking rats, which may contribute to the development of smoking-related inflammation in the lungs of rats because higher levels of NO can inhibit T cell function and induce cell apoptosis [25]. In contrast, we found that transfusion of BMSCs significantly mitigated the smoking-reduced serum and lung iNOS, which may contribute to therapeutic effect of BMSCs on smoking-induced lung inflammation. While a previous study reported that BMSCs can induce T cell arrest at G1 phase in mice [26], we did not observe a significant difference in the frequency of different phases of cycling cells among the different groups of rats. The discrepancy between our results and those from mice model [26] may stem from different disease models, animal ages, and experimental protocols. Hence, induction of T cell arrest by BMSCs transfusion may not be a mechanism underlying the action of BMSCs in our experimental model.

In this study, we employed a rat model of chronic cigarette exposure that endured for 24 weeks (initiate cigarette smoke exposure at 8 weeks old); it is for us to investigate whether the therapeutic effect of BMSCs on lung inflammation is specific for smoking-induced manner in the next experiment, including subacute exposure (for 4 weeks, initiate cigarette smoke exposure at 28 weeks old), subacute exposure (for 4 weeks, initiate cigarette smoke exposure at 8 weeks old), and so on.

## 5. Conclusions

Our data indicated that chronic smoking induced the lung inflammation and injury, which was attributed to the imbalance of proinflammatory and anti-inflammatory T cell responses in the lungs of rats. Transfusion of BMSCs improved the imbalance of proinflammatory and anti-inflammatory T cell responses and reduced anti-elastin responses, accompanied by enhanced iNOS expression in serum and lung and reducing STAT5 phosphorylation in lymphocytes from lung tissue. Hence, our data may provide new insights in the pathogenesis of smoking-induced lung inflammation in rats. Our findings suggest that BMSCs may be promising for the intervention of chronic smoking-related lung inflammation and COPD.

## Abbreviations

COPD: Chronic obstructive pulmonary disease
Th: T helper type
IFN-*γ*: Interferon-*γ*
IL: Interleukin
Foxp3: Forkhead box P3 (the Treg-defining transcription factor)
TNF-*α*: Tumor necrosis factor *α*
MLI: Mean linear intercept
MAN: Mean alveolar number.
